# A comparative evaluation of the efficacy between skeletal traction and skin traction in pre-operative management of femur shaft fractures in Korle Bu Teaching Hospital

**DOI:** 10.4314/gmj.v54i3.4

**Published:** 2020-09

**Authors:** Daniel Y D Agbley, Henry A Holdbrook-Smith, Yao Ahonon

**Affiliations:** 1 University of Health and Allied Sciences, Surgery, Ho Teaching Hospital, Ho; 2 Department of Trauma and Orthopaedics, Korle Bu Teaching Hospital, Korle Bu – Accra; 3 Public Health Unit, Korle Bu Teaching Hospital, Korle Bu, Accra

**Keywords:** Skin traction, Trans-tibia skeletal, reamed Intramedullary nailing, Intra-operative blood loss, Visual Analogue Scale

## Abstract

**Objectives:**

This study is to compare the outcomes of pre-operative skeletal and skin traction in adult femoral shaft fractures awaiting surgical fixation within two weeks of presentation to the Accident Center of Korle Bu Teaching Hospital.

**Methods:**

This study was a clinical trial on 86 recruited patients with closed femoral shaft fractures sustained within 24 hours of presentation grouped into 2 groups. Descriptive and inferential statistics comprising frequency, percentage, Chi-square, independent sample t-test and Mann-Whitney U test were used in analysing the data.

**Results:**

Of the total number of patients involved in the study, 74% (n=64) were males and 26% (n=22) were females with a mean age of 39.49 (SD ±15). There was no statistically significant difference in the mean visual analogue scale (VAS) pain assessment between the Skin traction group and Trans-tibia skeletal traction group after traction. With regards to complications, the difference between the Skin traction group and the Skeletal traction group was statistically significant (P=0.001). Moreover, the mean blood loss compared with the open type of reduction in the Transtibia skeletal traction group was significantly less than the Skin traction group (p=0.000).

**Conclusion:**

This study has shown that both Skeletal traction and Skin traction were equally effective in controlling pre-operative pain in adult patients with femoral shaft fractures and does not affect intra-operative blood loss and post-operative management. Therefore, pre-operative Skin traction can be considered a useful and cost-effective method of maintaining alignment and pain relief in adult femoral shaft fractures.

**Funding:**

Personal Funding

## Introduction

Fractures of the femoral shaft are among the most common fractures that orthopaedic surgeons encounter in Korle-Bu Teaching Hospital.^1^ traction is an important and basic treatment principle of orthopaedics. The use of traction devices to treat spinal injuries was described as early as 3000 to 2500 BC.^2^ The practice has since been adopted to treat nearly every fracture of the axial and appendicular skeleton. The treatment of femoral shaft fractures has evolved from the historical non-operative management to the most recent methods of intramedullary nail fixation.^3^–^6^ Traction has been the traditional method of treatment of femoral fractures, but this method has proved to have a high rate of malunion and knee stiffness, therefore, in recent times surgery has become the ideal treatment for these fractures.^5^

Skeletal traction and skin traction can be used to regain length of the injured limb. Ideally, skeletal traction applied to the distal femur or proximal tibia is balanced against counter traction by the body weight. Skin traction may be applied by way of adhesive tape, tapes bandaged to the limb or a traction boot^7^. Traction however, makes nursing of the patient more difficult: for example, in lifting the patient onto a bedpan or in pressure area care before surgery. Other possible adverse effects of skin traction are damage to the skin by mechanical shearing, ischaemia to the limb from tight bandages or allergy to adhesive strapping.

If skeletal traction is used with a tibia pin, the application of this can be uncomfortable, with the occasional complication of sepsis at the pin site.^5^

A few other studies done by Vanlaningham et al^4^ and Mao XH et al^8^ showed that there was no advantage in using skeletal traction over skin traction in the treatment of patients with closed fracture of the femur.

The treatment of femoral shaft fractures has evolved from the historical non-operative management to the most recent methods of intramedullary nail fixation^9^. Interlocking nails have greatly expanded the indications for closed intramedullary nailing of femoral fractures. Early mobilization following fractures of the femoral shaft has been shown to have a significant advantage in terms of both joint mobility and economic impact, which are very well attained by the use of interlocking nails^10^,^11^. Femoral shaft fractures are thus treated with skeletal traction with its known complications of prolonged bed rest and hospitalization, along with pin tract infections^9^,^12^, decubitus ulceration, malunion/non-union and shortening^10^.

Distal femoral skeletal traction remains a commonly used method for pre-operative fracture stabilization and pain control in patients with femoral shaft, neck, acetabular, and unstable pelvic fractures^13^. The purpose of this study was to compare the outcomes (pain, limb length discrepancy, intraoperative blood loss and complications) of preoperative Skeletal and Skin traction in adult femoral shaft fractures awaiting surgical fixation within 2 weeks of presentation in Korle Bu Teaching Hospital.

## Methods

### Patient selection and Data source

In this prospective randomized study, assuming a medium effect size (0.25) among the means of pain levels and its conforming Pearson correlation coefficient (0.3) value of repeated measurements and using a 80% power at 5% level of significance giving non-centrality parameter (10.2) which follows an F-distribution, 86 patients with femoral shaft fractures awaiting surgical fixation who presented to the Accident Centre of the Korle Bu Teaching Hospital from May to December 2017 were recruited.

The patients recruited for this study were randomized into two groups, and these groups were labelled A and B respectively and placed in an envelope. The researcher drew one randomly for each patient. Before the study, the patients or their companions were asked to fill in and sign the informed consent form in case of their willingness to participate in the study. In addition, the patients were assured that in case of severe pain, all possible measures would be taken for pain relief. The demographic data were collected with a structured data collection sheet.

The sheet captured the Age, Sex, fracture configuration (Transverse, Oblique or Spiral), Level of Fracture (Proximal, Middle or Distal Third).

A structured evaluation demographic form was administered to all consenting patients with femoral shaft fracture. Visual Analogue Scale Form (VAS) was administered and the scores documented.

### Assessment

Group A: Patients had skin traction applied at the Accident Center with an adhesive plaster on the skin of the thigh and the leg, wrapped around with bandage. A weight of 7 per cent of the patient's body of weight was attached. The skin traction kit was changed if it became defective.

Group B: Patients had trans-tibia skeletal traction done in the theatre under sterile condition. A 4.5 mm threaded Steinmann pin was passed from lateral to medial after local infiltration with 2% lidocaine with the awareness of the proximity of peroneal nerve, anterior tibia artery at the proximal tibia. The pin was inserted approximately 2.5 cm posterior and 2.5 cm distal to the tibia tubercle, parallel to the joint and a weight of 10 per cent of patient's body weight was attached. A Visual Analogue Scale (VAS) pain score was used to measure pain before and after traction. It is a measurement instrument consisting of a scale from 0 to10 cm in which zero indicates no pain, and ten indicates the worst pain imaginable.

The VAS pain score was used to assess the severity of pain in the patients at admission, 8-hourly for 24 hours after traction then daily before surgery and 8-hourly for 24 hours after surgery. Limb length discrepancy (LLD) was measured before the traction and preoperatively in the operating theatre. Intra-operatively, blood loss was assessed by weighing the wet swabs, blood on the floor was mopped with swabs and weighed. Blood loss in the suction container was recorded.

Post-operative complications, surgical site infection and total cost of treatment were assessed and documented. Post-operative limb length was measured and documented, and the patient followed up for two weeks. To control for performance biases in the study as a result of subjective assessment of outcomes by the surgeon, the investigator did a cluster stratification of patients, in which all patients having the procedure by one surgeon were placed into the same study group and the outcomes compared.

Cluster stratification of patients allowed surgeons to perform only the procedure with which they are most comfortable or experienced, providing a more valid assessment of the procedures being evaluated.

### Statistical Analysis

Data obtained was entered in Microsoft Excel 2016 and exported to Statistical Package for Social Sciences (IBM SPSS Version 25) for analysis. Continuous variables summarized as means (standard deviations). Categorical data were summarized as percentages, graphs and tables. Differences between the groups and the variables were examined using Chi-square test and the independent t-test or Mann-Whitney U test. P-value ≤ 0.05 was considered statistically significant.

### Ethics

The study was reviewed and approved by The Research and Ethical Review Committee of the College of Health Sciences and written informed consent was obtained from the patients or wards before the investigation (Date of issue: August 28, 2017; protocol identification number: CHS-Et/M.10 – P3.6/2016-2017). Participants were assured of anonymity and confidentiality of the information provided.

## Results

Of the total number of patients involved in the study (n=86), 74% were males, 26% were females with a mean age of 39.5 years (SD ±15.0). The mean age of the Skin traction group was 39.2 years (SD ±15.0) and Trans-tibia skeletal traction group was 39.8 years (SD ±15.2). In the Skin traction group, the etiological factors were Road traffic accident (67.4%), fall from a height (18.6%), direct blow (9.4%), sports injury (2.3%) and other forms (2.3%). In the Trans-tibia skeletal traction group, the aetiology of the injuries was road traffic accident (88.3%), fall from height (4.7%), direct injury (0.0%), sports injury (2.3%) and other forms (4.7%).

There was a statistically significant difference in the various injury etiologies between the Skin traction group and the Skeletal traction group (p=0.02). There was no statistical difference in the side of fracture between the two groups (p= 0.829). The level of fracture in the Skin traction group revealed that 18 (41.9%) were middle 1/3, 16 (37.2%) were proximal 1/3, and 9 (20.9%) were distal 1/3. In the Tans-tibia skeletal traction group, 19 (44.2%) were middle 1/3, 15 (34.9%) were proximal 1/3, and 9 (20.9%) were distal 1/3. The groups did not differ statistically regarding age group, sex, location of injury, side of fracture and level of fracture (p>0.05 for all) (Table 1).

**Table 1:** Demographic data of the participant in the study

Variables		Skin Traction (n=43)	Skeletal Traction (n=43)	P-value
**Age**	Mean (SD)	39.2(15.0)	39.8(15.2)	0.839
**Gender**	Male	33	31	0.805
	Female	10	12	
**Mechanism of** **Injury**	RTA	29	38	*0.026
	Fall from Height	8	2	
	Direct blow	4	0	
	Sports injury	1	1	
	Others	1	2	
**Side of fracture**	Left femur	24	22	0.829
	Right Femur	19	21	
**Level of Fracture**	Distal 1/3	9	9	0.09
	Middle 1/3	18	19	
	Proximal 1/3	16	15	

* Statistically significant (p<0.05)

The mean shortening before traction was 2.91cm (SD ±1.1) in the Skin traction and 3.23cm (SD ±1.0) in the Trans-tibia skeletal traction group, however, there was no significant difference (P>0.05). The mean shortening before surgery was 1.3cm (SD ±0.5) in the Skin traction and 1.2cm (SD ±0.5) in the Trans-tibia skeletal traction group, no significant difference was found. The mean blood loss in the closed type of reduction in the Skin traction group 202.9ml (SD ±66.3) and the Trans-tibia skeletal traction group was 195.8ml (SD ±39.6). There was no statistically significant mean difference between the two groups (Table 2).

**Table 2:** Outcomes of interventions

Outcomes	Skin Traction (n=43)	Skeletal Traction (n=43)	P- value
	Mean (SD)	Mean (SD)	
**Shortening before Traction (cm)**	2.9 (1.1)	3.2 (1.0)	0.16
**Shortening before Surgery (cm)**	1.3 (0.5)	1.2 (0.5)	0.279
**Intra-operative blood loss (ml)**	202.9 (66.3)	195.8 (39.6)	0.085

From Table 3, the mean VAS pain score before traction was 6.7 (SD ±0.7) for both the Skin traction group and the Trans-tibia skeletal traction group.

**Table 3:** Comparison of the mean VAS of patients in two group in the different time points

VAS Scores	Skin Traction (n=43)	Skeletal Traction (n=43)	p-value
	Mean (SD)	Mean ± SD	
**VAS score pain before Traction**	6.7 (0.7)	6.7 (0.7)	1.000
**VAS score pain After Traction**	1.5 (0.8)	1.6 (0.7)	0.304^a^
**VAS score pain after Surgery**	0.8 (0.4)	1.1 (0.4)	*0.012
	**Median** **(IQR)**	**Median** **(IQR)**	
**VAS score pain after surgery** **(8hrs)**^c^	1.00	1.00 (1–2)	0.155^a^
**VAS score pain after surgery** **(16hrs)**^c^	1.00	1.00	1.000^a^
**VAS score pain after surgery** **(24hrs)**^c^	1.00	1.00	1.000^a^

* Statistically significant (p<0.05)

a Mann-Whitney U test

c Median score

The mean VAS pain score after traction was 1.5 (SD 0.8) for Skin traction group and 1.6 (SD ±0.7) for Trans-tibia skeletal traction group. The mean VAS pain score of the traction application after the surgery was higher in the Trans-tibia skeletal traction (1.1, SD ±0.4) compared to the Skin traction group (0.8, SD ±0.4) (p=0.012).

The Mann-Whitney U test indicated that there was no statistically significant difference in the median VAS pain score of the two groups at 8 hours, 16 hours and 24 hours of traction application as shown in Table 3. None of the patients in the Skin traction group had any complications and in the Trans-tibia skeletal traction group, 10 patients (23.3%) had pin tract infection, this difference was statistically significant (p=0.001) (Table 4).

**Table 4:** Complications of interventions

Complications	Skin Traction (n=43)	Skeletal Traction (n=43)	Total	P-value
**Yes**	0	10	10	*0.001
**No**	43	33	76	
**Total**	43	43	86	

* Statistically significant (p<0.05)

## Discussion

Femoral shaft fractures in adults are reasonably common and can occur in isolation or in association with other injuries. They are typically high-energy in etiology, especially in young adults, and require special consideration to the physiological impact of injury on the patient. The evolution of treatment for these fractures has seen changes in the timing of surgery as well as the techniques employed, but the principles of stable internal fixation remain unchanged.

Good outcomes and low complication rates can be expected if the operating surgeon has a thorough understanding of the anatomy, basic science and surgical technique relating to the treatment of femoral shaft fractures.

Skeletal traction has been used in the past for the management of femoral shaft fractures in adults and skin traction in paediatric femoral shaft fractures, but the method was associated with complications such as pin tract infections, bedsores and hypostatic pneumonia in adults. Patients have to be in bed for more than six weeks for the fracture to heal, and the majority end up with mal-union, non-union and leg length discrepancies. With the considerable advances in the discovery of newer orthopaedic implants such as intramedullary nails, locked plates, most adult patients with femoral shaft fractures are being treated early surgically.

The study demonstrated that femoral shaft fractures occurred mostly in the male gender accounting for 76% of the studied population as against 24% female gender. Most of these femoral shaft fractures occurred as a result of a road traffic accident. This finding confirms the fact that the major risk-takers on our roads are the male gender.

This study had shown that the application of skin traction resulted in a decrease in pain intensity on the Visual Analogue Scale (VAS) from 7 in most patients to 1 or 2 after admission. In both study groups, pain reduced from 7 to 1 in most patients. Post-operatively pain had been consistently 1 on VAS after 8 hours, 16 hours and 24 hours. This finding is consistent with a study done by Alireza Manafi Rasi et al., that concluded that traction does not interfere with the need for analgesics, but it remarkably decreases the pain in patients with proximal femur fractures and it assists the patient's comfort and relaxation.^13^ The application of skin traction before the surgery in these patients is recommended.

Shortening is frequently observed due to the pull of the hamstrings and quadriceps muscles which act as deforming forces after a fracture. In proximal fractures (in the subtrochanteric region), the proximal segment is typically flexed, abducted, and externally rotated by the muscular pull of the hip abductors, external rotators, and iliopsoas^5^. Shortening before traction in the study population with femoral shaft fracture was between 1cm and 5 cm. Shortening before surgery in the skin traction group revealed that, 30 patients had a residual shortening of 1cm while 12 patients had residual shortening of 2 cm before surgery. In the skeletal traction group, 37 patients had residual shortening of 1cm before surgery, and only seven patient had 2 cm residual shortening before surgery.

Comparing intraoperative blood loss in both study groups who had open reduction and fixation for femoral shaft fractures, there was a statistically significant mean difference (p<0.05). This finding did not translate into increase blood transfusion requirement and is consistent with a study done by Kajja et al.^14^

In this study, there was no single demonstrable skin allergy to plaster in the skin traction group while in the Skeletal traction group, 10 (23.3%) patients developed superficial pin tract infection. This finding is in accordance with the study done by Gosselin RA, Heitto M, Zirkle L. and Musajee M that concluded that although simple, the insertion and care of these pins can be accompanied by severe complications including damage to neurovascular structures, ligamentous insult, fracture, and infection.^9^,^13^

## Conclusion

This study has shown that both Skeletal traction and Skin traction were equally effective in controlling pre-operative pain in adult patients with femoral shaft fractures and does not affect intra-operative blood loss and post-operative management. Pre-operative skin traction can be considered a suitable and cost-effective method of maintaining alignment and pain relief in adult femoral shaft fractures

## References

[1] Korle Bu Teaching Hospital (2012). Annual Report.

[2] Matullo KS, Gangavalli A, Nwachuku C (2016). Review of Lower Extremity Traction in Current Orthopaedic Trauma. J Am Acad Orthop Surg.

[3] Even JL, Richards JE, Crosby CG, Kregor PJ, Mitchell EJ, Jahangir AA, Tressler MA, Obremskey WT (2012). Pre-operative skeletal versus cutaneous traction for femoral shaft fractures treated within 24 hours. J Orthop Trauma.

[4] Vanlaningham CJ, Schaller TM, Wise C (2009). Skeletal versus skin traction before definitive management of pediatric femur fractures: a comparison of patient narcotic requirements. J Pediatr Orthop.

[5] Parker M, Handoll H (2003). Pre-operative traction for fractures of the proximal femur in adults. Cochrane Database of Syst Rev.

[6] Access O, Opondo E, Wanzala P, Makokha A (2013). Cost effectiveness of using surgery versus skeletal traction in management of femoral shaft fractures at Thika level 5 hospital, Kenya. Pan Afr Med J.

[7] Skin Traction and Skeletal Traction. Bone and Spine.

[8] Mao Mao XH, Yan J (2014). [Effect of pre-operative skeletal traction and skin traction on operative indicators and functional outcome of patients with femur fractures]. Zhongguo gu shang= China journal of orthopaedics and traumatology.

[9] Gosselin RA, Heitto M, Zirkle L (2009). Cost-effectiveness of replacing skeletal traction by interlocked intramedullary nailing for femoral shaft fractures in a provincial trauma hospital in Cambodia. Int Orthop.

[10] Gosselin R, Lavaly D (2007). Perkins traction for adult femoral shaft fractures: A report on 53 patients in Sierra Leone. Int Orthop.

[11] Deepak MK, Jain K, Rajamanya KA, Gandhi PR, Rupakumar CS Functional outcome of diaphyseal fractures of femur managed by closed intramedullary interlocking nailing in adults. Ann Afr Med.

[12] Musajee M (2012). Outcome of skeletal traction in patients with femoral shaft fractures at Kenyatta National hospital.

[13] Manafi Rasi A, Amoozadeh F, Khani S, Kamrani Rad A, Sazegar A (2015). The Effect of Skin Traction on Pre-operative Pain and Need for Analgesics in Patients with Intertrochanteric Fractures: A Randomized Clinical Trial. Arch Trauma Res.

[14] Kajja I, Bimenya GS, Eindhoven B, Ten Duis HJ, Sibinga CT (2010). Blood loss and contributing factors in femoral fracture surgery. Afr Health Sci.

